# Social support as a moderator between birth satisfaction and breastfeeding self-efficacy among polish mothers

**DOI:** 10.3389/fgwh.2026.1813521

**Published:** 2026-05-29

**Authors:** Agnieszka Czerwińska-Osipiak, Anna Weronika Szablewska, Krzysztof Jurek, Conceição Santiago

**Affiliations:** 1 Department of Obstetric and Gynaecological Nursing, Institute of Nursing and Midwifery, Faculty of Health Sciences with the Institute of Maritime and Tropical Medicine, Medical University of Gdańsk, Gdańsk, Poland; 2 Institute of Sociological Sciences, Faculty of Social Sciences, John Paul II Catholic University of Lublin, Lublin, Poland; 3 Higher School of Health, Santarém Polytechnic University, Santarém, Portugal; 4 Research and Innovation in Health—RISE-Health, Faculty of Medicine, University of Porto, Porto, Portugal

**Keywords:** birth satisfaction, breastfeeding self-efficacy, lactation, maternal health, social support, socioeconomic factors

## Abstract

**Background:**

Breastfeeding self-efficacy is one of the psychological determinants of breastfeeding initiation and duration. Although both birth satisfaction and social support have been associated with breastfeeding outcomes, their combined effects on breastfeeding self-efficacy remain poorly explored.

**Objective:**

The aim of this study was to investigate the relationships between birth satisfaction, perceived social support and breastfeeding self-efficacy among Polish mothers, with particular focus on the moderating role of social support.

**Methods:**

In this cross-sectional study, 705 Polish mothers who had given birth within the past 24 months, reporting any form of breastfeeding, were included. The participants completed the online Breastfeeding self-Efficacy Scale-Short Form questionnaire, the Birth Satisfaction Scale-Revised, and the Multidimensional Scale of Perceived Social Support. Moderation analysis was conducted using linear regression with interaction terms, controlling for maternal age, parity, partner encouragement, pregnancy complications, skin-to-skin contact and mode of birth.

**Results:**

Birth satisfaction and perceived social support were independently associated with higher breastfeeding self-efficacy. A significant interaction effect was observed, indicating that perceived social support moderated the relationship between birth satisfaction and breastfeeding self-efficacy. Simple slopes analysis showed that this association weakened as social support increased and became negative at high levels of support. Importantly, no statistically significant correlations were observed between birth satisfaction and breastfeeding self-efficacy at low levels of perceived social support, whereas at its mean and high levels, the association was negative and statistically significant.

**Conclusion:**

The study findings suggest that perceived social support plays a compensatory role in shaping breastfeeding self-efficacy, reducing reliance on birth satisfaction when support is high. In the absence of strong social support, birth satisfaction alone does not appear to be significantly associated with breastfeeding self-efficacy. Strengthening social support systems, particularly for women with less positive birth experiences, may enhance breastfeeding confidence and contribute to improved breastfeeding outcomes.

## 1 Introduction

Breastfeeding is widely recognized as a public health priority due to its well-established benefits for both maternal and infant health (1, 2). Despite clear international recommendations advocating exclusive breastfeeding for the first six months of life, followed by continued breastfeeding alongside complementary feeding, adherence to these guidelines remains suboptimal in many countries (3, 4). A substantial proportion of women discontinue breastfeeding earlier than recommended, often within the first weeks or months postpartum (5). At the same time, the progress noted towards the 2025 global nutrition targets provides a foundation for further efforts to achieve the aim for 2030: to increase the rate of exclusive breastfeeding in the first six months up to at least 60% (6).

In previous research, it has been consistently indicated that breastfeeding self-efficacy—defined as a mother's confidence in her ability to breastfeed successfully—is a central psychological determinant of breastfeeding initiation, duration and exclusivity (7). Higher breastfeeding self-efficacy has been previously associated with longer breastfeeding duration, lower risk of early cessation and greater persistence in the face of lactation difficulties (8–10). Moreover, identifying factors that shape breastfeeding self-efficacy is important for designing effective interventions to support breastfeeding practices (11–13).

Among the potential determinants of breastfeeding self-efficacy, both birth satisfaction and social context appear particularly relevant. Higher birth satisfaction has been linked to enhanced maternal confidence, emotional well-being and early mother–infant bonding, all of which may influence subsequent breastfeeding behaviors (14). In previous studies, it has been demonstrated that mothers reporting more positive birthing experiences, including higher levels of birth satisfaction, are more likely to initiate and sustain breastfeeding for longer durations, whereas negative or traumatic birth perceptions are associated with poorer maternal well-being and less favorable breastfeeding outcomes (15, 16). Higher birth satisfaction has also been related to more positive breastfeeding attitudes, suggesting that subjective evaluation of childbirth may play an important role in shaping early feeding practices (16). Similarly, perceived social support—especially from partners, family members and healthcare professionals—has been shown to facilitate breastfeeding by providing emotional reassurance, practical assistance and encouragement (17–19). However, in the majority of existing studies, these factors have been examined independently, and relatively little is known about how birth satisfaction and social support interact in shaping breastfeeding self-efficacy.

Such a perspective closely aligns with the self-efficacy theory proposed by Albert Bandura. self-efficacy—first conceptualized within social cognitive theory—is defined as an individual's belief in their ability to succeed in a specific situation. This belief is crucial in determining behavior as it reflects a person's perception of his/her contextual abilities (20). This theory posits that beliefs about personal competence arise from a dynamic interplay between individual experiences and environmental influences (21). Applied to this context, Dennis (22) developed the breastfeeding self-efficacy theory, defining it as a mother's confidence in her perceived ability to breastfeed her infant. According to this framework, self-efficacy is primarily shaped by mastery experiences (direct personal successes or failures), but it is also influenced by social persuasion and supportive feedback. Dennis further identifies four key sources influencing this confidence: mastery experiences (past experiences), vicarious experiences, verbal persuasion, and physiological responses. Consequently, mothers with higher breastfeeding self-efficacy are more likely to initiate nursing, persist despite challenges and respond to difficulties in a positive manner (6, 23).

From this standpoint, childbirth may represent a mastery experience for new mothers, while social support constitutes a contextual resource that can reinforce, or compensate for, these experiences. Moreover, Bandura's theory suggests that the impact of personal experiences on self-efficacy is not fixed but may vary depending on the availability of external support (21). Despite the relevance of this theoretical framework, empirical research examining the joint effects of birth satisfaction and perceived social support on breastfeeding self-efficacy remains limited. In particular, it is unclear whether social support amplifies the positive impact of birth satisfaction or, conversely, creates a buffer against less optimal experiences by providing alternative sources of confidence and reassurance. Addressing this gap is especially important in contexts where breastfeeding rates remain below recommended levels and psychosocial determinants of breastfeeding have been insufficiently explored.

Therefore, the aim of the present study was to investigate the relationships between birth satisfaction, perceived social support and breastfeeding self-efficacy among Polish mothers, with specific focus on the moderating role of social support. By integrating these variables within a single analytical model, the objective of the present research is to extend existing knowledge on the psychosocial foundations of breastfeeding confidence. Understanding how individual birth experiences interact with social resources may inform more targeted perinatal and postnatal interventions, highlighting the significance of both supportive childbirth practices and strengthened social support systems in promoting sustained breastfeeding.

## 2 Materials and methods

### 2.1 Study design and participants

This observational cross-sectional study was conducted among Polish mothers who had given birth within the past 24 months and reported engaging in any type of breastfeeding after their most recent delivery. A total of 705 breastfeeding women were included in the final sample after exclusion of inconsistent or contradictory responses. A nationwide sample of Polish mothers was recruited via online platforms and social media groups dedicated to motherhood. The inclusion criteria required the child to be ≤24 months old and that the mother had breastfed after the last childbirth or was currently breastfeeding. Women who delivered pre-term or who exclusively formula-fed their infants during the first six months were excluded. These exclusion criteria were applied to maintain a relatively homogeneous study population and to focus on breastfeeding self-efficacy among mothers with breastfeeding experience after term birth. Pre-term birth is associated with distinct clinical and lactation-related circumstances, including possible neonatal immaturity, NICU admission, maternal–infant separation, delayed or interrupted skin-to-skin contact, and various patterns of professional lactation support. Mothers who exclusively formula-fed during the first six months were also excluded because their infant-feeding trajectories may reflect different medical, psychological, social or structural determinants that were beyond the scope of the present analysis. Screening questions regarding child age and feeding method were included in the questionnaire and served to automatically exclude individuals who did not meet the eligibility criteria. All participants provided informed consent prior to participation. Ethical approval was obtained from the Bioethics Commission of the Medical University of Gdańsk (NKBBN/19/2025). All procedures were conducted in accordance with the principles of the 1964 Declaration of Helsinki (and its later amendments), an internationally recognized set of ethical principles for medical research involving human subjects, encompassing requirements for informed consent, privacy protection and the right to withdraw at any time. The sample size provided sufficient statistical power to detect moderate effect sizes in multiple regression analyses with eight predictors. The observation-to-predictor ratio was approximately 88:1, exceeding the recommended thresholds and supporting stability of parameter estimates. Missing data were minimal, addressed using conditional group-mean imputation. Given the small share of missing values and the large sample size, the impact of imputation on the results is likely to be limited. However, this approach may influence variance estimates, which is acknowledged as a limitation.

### 2.2 Data collection

Data were collected using an anonymous self-administered online questionnaire between May and October 2025. The questionnaire was developed by a multidisciplinary team including a midwife and two certified lactation consultants.

The collected data included socio-demographic and perinatal characteristics (maternal age, education, place of residence, self-assessed financial and housing conditions, parity, mode of delivery, partner encouragement towards breastfeeding, pregnancy complications and skin-to-skin contact after birth), as well as infant feeding practices (exclusive breastfeeding, mixed feeding, formula feeding).

For measuring breastfeeding self-efficacy, the Polish adaptation of the Breastfeeding self-Efficacy Scale–Short Form (BSES-SF) was used. This is a 14-item instrument rated on a five-point Likert scale, with higher scores indicating greater self-efficacy (Cronbach's *α* = 0.89 in the Polish population) (24, 25). Although originally validated in the early postpartum period, the BSES-SF has subsequently been tested both antenatally and up to 12 months postpartum. It has received a Grade A recommendation for use across the continuum of maternity care (23, 26) and has been applied in studies of breastfeeding mothers of infants up to 24 months of age and beyond (27, 28). Perceived social support was assessed via the Multidimensional Scale of Perceived Social Support (MSPSS), adapted for Polish populations. This scale consists of 12 items rated on a seven-point Likert scale (Cronbach's *α* > 0.90) (29).

Birth satisfaction was evaluated using the Birth Satisfaction Scale–Revised (BSS-R), a 10-item measure rated on a five-point Likert scale. Only the total score was used in the analyses. The Polish version demonstrates good reliability (Cronbach's *α* = 0.79–0.87) (30, 31).

### 2.3 Variables

Standardized and validated instruments were used for all psychometric measures:

- Dependent variable (Y): Breastfeeding self-efficacy (BSES-SF total score);
- Independent variable (X): Birth satisfaction (BSS-R total score);
- Moderator (M): Perceived social support (MSPSS total score);
- Interaction term (X × M), computed using mean-centered BSS-R and MSPSS scores—included to test moderation.

The conceptual model assumes a moderating relationship, whereby perceived social support (M) conditions the association between birth satisfaction (X) and breastfeeding self-efficacy (Y). In such a framework, the effect of X on Y is not constant but changes depending on the level of M. In practice, the moderating variable can strengthen, weaken or even reverse the direction of the predictor's effect on the outcome, allowing for a more precise understanding of the mechanisms underlying the analyzed relationship.

Control variables were selected based on indications in prior literature and included maternal age, number of children, mode of birthing, partner encouragement towards breastfeeding, pregnancy complications and skin-to-skin contact after birth. In the regression model, maternal age was treated as a continuous variable. Primiparity was coded as 1 for first-time mothers and 0 for multiparous women. Partner's encouragement towards breastfeeding, pregnancy complications, uninterrupted skin-to-skin contact during the first two hours after birth, and mode of birth were entered as categorical control variables according to the coding used in the regression model.

### 2.4 Statistical analysis

In the performed statistical analysis, categorical variables are presented as percentages (%) and numbers (n). Quantitative variables with normal distribution are presented as means (M) and standard deviations (SD).

Pearson's correlation coefficient (Pearson's r) was used to evaluate the relationship between the studied variables. A bias-corrected bootstrap estimation (5,000 samples) with a confidence interval of 95% was applied to evaluate the moderating role of social support (MSPSS) between self-assessed breastfeeding effectiveness (BSES-SF) and birth satisfaction (BSS-R). Prior to conducting regression analyses, standard assumptions were assessed. Linearity and homoscedasticity were evaluated through visual inspection of residual plots. Normality of residuals was assessed using histogram and Q–Q plots. Multicollinearity was examined via variance inflation factors (VIF), which remained below commonly accepted thresholds. No influential outliers were identified based on Cook's distance. For the analysis, SPSS 29 statistic software and PROCESS (model 1) for SPSS were used. An *a priori* power analysis was conducted implementing G*Power for multiple linear regression. Assuming a medium effect size (f^2^ = 0.15), *α* = 0.05, powe*r* = 0.80 and nine predictors (including the interaction term), the required sample size was *N* = 114. The achieved sample size (*N* = 705) exceeded this threshold and provided sufficient power to detect even small effects.

## 3 Results

### 3.1 Study group characteristics

In the study, the mean age of the participants was 32.11 years (SD = 4.88). The majority of women reported having higher education (83.1%), followed by secondary (15.5%); and only a small proportion had vocational (1.1%) or primary education (0.3%). Nearly half of participants lived in cities with more than 100,000 inhabitants (46.1%), while 27.4% resided in rural areas and 26.5% in smaller cities.

As for financial situation, majority of women rated theirs as good (58.6%) or very good (26.7%), and housing conditions were similarly evaluated, with 48.7% reporting very good and 40.6% good conditions. Previous childbirths were reported by 61.7% of participants, whereas 38.3% were primiparous.

Partner encouragement towards breastfeeding was reported by 70.6% of women, 12.3% indicated limited support, 11.3% reported no support, and 5.6% selected “not applicable”. Pregnancy complications or pre-existing medical conditions were reported by 34.3% of participants. Continuous two-hour skin-to-skin contact after birth occurred in 68.9% of cases; interruptions were attributed to maternal health (8.9%), infant health (8.8%) or unspecified reasons (13.4%).

The mean breastfeeding self-efficacy score (BSES-SF) was 58.00 (SD = 9.23). Perceived social support (MSPSS) was relatively high (M = 64.59; SD = 14.50), and the mean birth satisfaction score (BSS-R) was 22.96 (SD = 7.81). The sociodemographic and clinical characteristics of the sample are presented in Table 1.

**Table 1:** Sociodemographic, perinatal and psychometric characteristics of studied sample (*N* = 705).

Characteristic		M/N	SD/%
Age		32.11	4.88
Education level	Primary	2	.3
	Vocational	8	1.1
	Secondary	109	15.5
	Higher (Bachelor's/Master's degree)	586	83.1
Place of residence	Village	193	27.4
	City < 100k residents	187	26.5
	City > 100k residents	325	46.1
Financial situation	Very good	188	26.7
	Very bad	1	.1
	Good	413	58.6
	Satisfactory	101	14.3
	Bad	2	.3
Housing conditions	Very good	343	48.7
	Good	286	40.6
	Satisfactory	73	10.4
	Bad	3	.4
Parity	Primiparous	270	38.3
	Multiparous	435	61.7
Mode of birth	Cesarean section	264	37,4
	Vaginal	441	62,6
Partner's encouragement towards breastfeeding	Not applicable	40	5.6
	No encouragement	80	11.3
	Limited encouragement	87	12.3
	Full encouragement	498	70.6
Pregnancy complications	None	463	65.7
	Yes	242	34.3
Uninterrupted skin-to-skin contact during first two hours after birth	No, it was interrupted without reason	94	13.4
	No, due to maternal health	63	8.9
	No, due to infant health	62	8.8
	Yes, it was maintained	486	68.9
Breastfeeding Self-Efficacy Score (BSES-SF)		58.00	9.23
Perceived Social Support Score (MSPSS)		64.59	14.50
Birth Satisfaction Score (BSS-R)		22.96	7.81

Data presented as mean (standard deviation) for continuous variables and *n* (%) for categorical variables.

### 3.2 Outcomes

Correlation analysis **(**Table 2**)** showed that breastfeeding self-efficacy (BSES-SF) was weakly but significantly negatively correlated with birth satisfaction (BSS-R) (*r* = −0.089, *p* = 0.018), indicating that higher levels of birth satisfaction were associated with slightly lower breastfeeding self-efficacy. Perceived social support (MSPSS) demonstrated a significant, weak positive correlation with birth satisfaction (BSS-R) (*r* = 0.118, *p* = 0.002), suggesting that higher perceived social support co-occurred with higher levels of birth satisfaction. No significant correlations were observed between perceived social support and breastfeeding self-efficacy (*r* = −0.004, *p* = 0.915).

**Table 2:** Correlations between breastfeeding self-efficacy (BSES-SF), perceived social support (MSPSS) and birth satisfaction (BSS-R).

Variable	BSES-SF	MSPSS	BSS-R
BSES-SF	1.000		
MSPSS	−0.004 (*p* = 0.915)	1.000	
BSS-R	−0.089* (*p* = 0.018)	0.118* (*p* = 0.002)	1.000

BSES-SF, Breastfeeding Self-Efficacy Scale-Short Form; MSPSS, Multidimensional Scale of Perceived Social Support; BSS-R, Birth Satisfaction Scale-Revised.

* *p* < 0.05.

**Figure 1:**
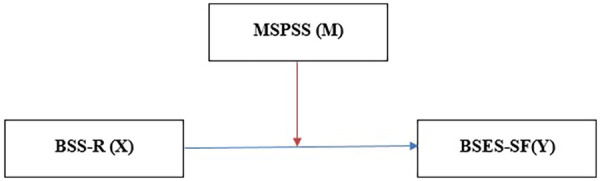
Moderating effect of perceived social support (MSPSS) on relationship between birth satisfaction (BSS-R) and breastfeeding self-efficacy (BSES-SF). Lines represent simple slopes at low (−1 SD), mean and high (+1 SD) levels of MSPSS.

Linear regression analysis incorporating an interaction term was performed to examine the relationships among breastfeeding self-efficacy (BSES-SF), birth satisfaction (BSS-R) and perceived social support (MSPSS). The conceptual moderation model illustrating the hypothesized relationships among variables is presented in Figure 1.

The regression model included birth satisfaction, perceived social support, the interaction term and the prespecified control variables. The overall model was statistically significant and explained a small but significant share of the variance in breastfeeding self-efficacy (*R*^2^ = 0.029, *F* = 2.20, *p* = 0.020). Detailed regression results are shown in Table 3. Significant positive main effects were observed for both birth satisfaction (BSS-R; *b* = 0.42, SE = 0.20, *p* = 0.039) and perceived social support (MSPSS; *b* = 0.19, SE = 0.07, *p* = 0.008), indicating that higher levels of both variables were associated with higher breastfeeding self-efficacy (BSES-SF).

**Table 3:** Linear regression results for breastfeeding self-efficacy (BSES-SF) with birth satisfaction (BSS-R), social support (MSPSS) and control variables.

Variable	B	SE	t	*p*	95% CI LL	95% CI UL
Constant	43.628	5.492	7.943	<0.001	32.844	54.413
Birth satisfaction (BSS-R)	0.417	0.201	2.069	0.039	0.021	0.812
Perceived social support (MSPSS)	0.188	0.070	2.684	0.008	0.050	0.325
BSS-R × MSPSS interaction	−0.008	0.003	−2.687	0.007	−0.014	−0.002
Age	0.088	0.079	1.121	0.263	−0.067	0.243
Primiparous (yes=1)	−0.498	0.770	−0.647	0.518	−2.010	1.013
Partner's encouragement toward breastfeeding (yes=1)	0.971	1.099	0.884	0.377	−1.186	3.128
Pregnancy complications (yes=1)	−0.374	0.748	−0.500	0.617	−1.843	1.095
Uninterrupted skin-to-skin contact during first two hours after birth (yes=1)	1.362	0.790	1.723	0.085	−0.190	2.914
Vaginal birth	0.172	0.764	0.225	0.822	−1.328	1.671

Model *R*^2^ = 0.029; F = 2.20; *p* = 0.020. Continuous predictors were mean-centered. Reference categories: Multiparous (for primiparous); No encouragement/Not applicable (for partner's encouragement); No (for pregnancy complications and skin-to-skin contact); Cesarean section (for vaginal delivery).

A statistically significant interaction between BSS-R and MSPSS was also identified (*b* = −0.0079, SE = 0.0029, *p* = 0.007). Although the additional variance explained by the interaction term was small (ΔR^2^ = 0.0107), the effect was considered reliable given the large sample size, indicating that the association between birth satisfaction and breastfeeding self-efficacy varied across levels of perceived social support. This suggests that when interpreting the main effect of birth satisfaction, perceived social support should be taken into account.

Maternal age was not a significant predictor of breastfeeding self-efficacy (*b* = 0.09, SE = 0.08, *t* = 1.12, *p* = 0.263). Similarly, parity (first vs. subsequent child) showed no significant effect (*b* = −0.50, SE = 0.77, *t* = −0.65, *p* = 0.518), and partner encouragement towards breastfeeding—although positive—did not reach the level of statistical significance (*b* = 0.97, SE = 1.10, *t* = 0.88, *p* = 0.377). Pregnancy complications (*b* = −0.37, SE = 0.75, *t* = −0.50, *p* = 0.617) and mode of birth (vaginal vs. cesarean section; *b* = 0.17, SE = 0.76, *t* = 0.22, *p* = 0.822) were also not significantly correlated with breastfeeding self-efficacy.

Skin-to-skin contact after birth showed a trend towards statistical significance (*b* = 1.36, SE = 0.79, *t* = 1.72, *p* = 0.085), indicating a potentially beneficial effect of early mother–infant contact on breastfeeding self-efficacy, although this association did not reach conventional levels of statistical significance.

**Figure 2:**
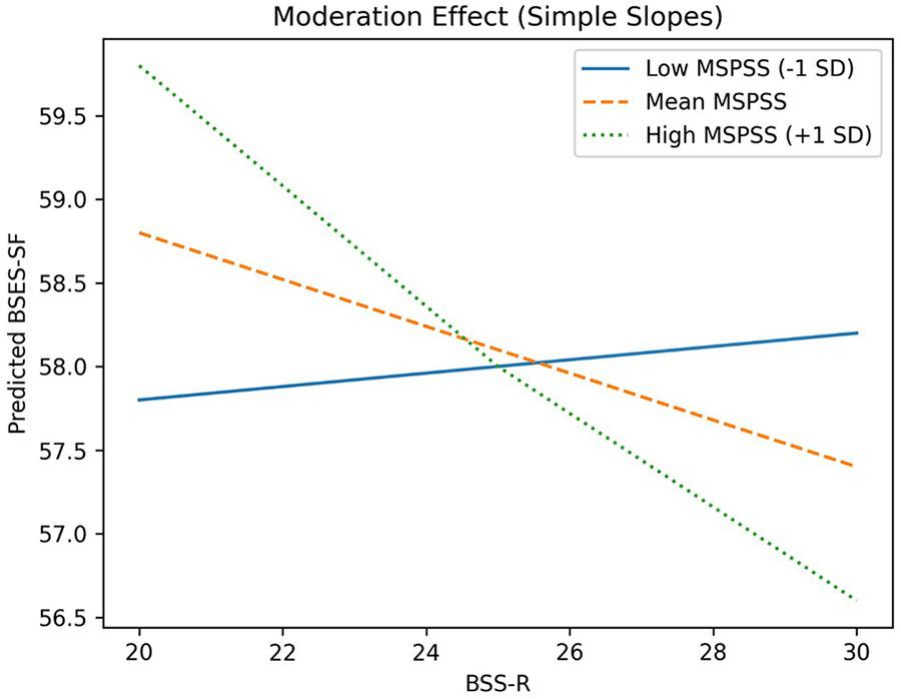
Simple slopes illustrating moderating effect of perceived social support (MSPSS) on relationship between birth satisfaction (BSS-R) and breastfeeding self-efficacy (BSES-SF).

Simple slopes analysis demonstrated that at low levels of perceived social support (−1 SD), the association between birth satisfaction and breastfeeding self-efficacy was positive but weak, and did not show statistical significance (*b* = 0.02, *p* = 0.729) (please see Table 4 and Figure 2). At the mean level of social support, this relationship reversed and reached statistical significance, with higher birth satisfaction associated with lower breastfeeding self-efficacy (*b* = −0.09, *p* = 0.048). This pattern was more pronounced at high levels of perceived social support (+1 SD), where the association between birth satisfaction and breastfeeding self-efficacy was clearly negative and statistically significant (b = −0.21, *p* < 0.001). Overall, increasing levels of perceived social support were associated with a progressively weaker—and ultimately negative—relationship between birth satisfaction and breastfeeding self-efficacy.

**Table 4:** Conditional effects of birth satisfaction (BSS-R) on breastfeeding self-efficacy (BSES-SF) at different levels of perceived social support (MSPSS).

Level of perceived social support (MSPSS)	B	SE	t	*p*	95% CI LL	95% CI UL
−1 SD (low support)	0.023	0.068	0.346	0.729	−0.109	0.156
M (moderate support)	−0.092	0.046	−1.980	0.048	−0.182	−0.001
+1 SD (high support)	−0.207	0.058	−3.554	< 0.001	−0.321	−0.093

B, unstandardized regression coefficient; SE, Standard error; CI, Confidence interval.

Higher birth satisfaction was associated with higher breastfeeding self-efficacy, and higher perceived social support was also connected with higher breastfeeding self-efficacy. However, the relationship between birth satisfaction and breastfeeding self-efficacy was not uniform across levels of perceived social support. As perceived social support increased, the strength of this association progressively decreased and became negative at high levels of support.

These findings indicate that no statistically significant association between birth satisfaction and breastfeeding self-efficacy was observed at low levels of perceived social support. In contrast, the correlation became negative and statistically significant at mean and high levels of support. This suggests that the relationship between birth satisfaction and breastfeeding self-efficacy is conditional upon the level of perceived social support, becoming increasingly negative as support increases.

The weak negative bivariate correlation contrasts with regression findings, underscoring that the relationship between birth satisfaction and breastfeeding self-efficacy is not linear or uniform but depends on contextual factors, particularly perceived social support.

## 4 Discussion

Associations between birth satisfaction, perceived social support and breastfeeding self-efficacy among Polish breastfeeding mothers were examined in the present study. The main finding was that both birth satisfaction and perceived social support were independently correlated with higher breastfeeding self-efficacy, and that perceived social support significantly moderated this relationship. Specifically, the association between birth satisfaction and breastfeeding self-efficacy weakened as levels of perceived social support increased and became negative at high levels of support. These results suggest that social support plays an important role in shaping maternal breastfeeding confidence and modifies the impact of birth satisfaction on breastfeeding self-efficacy.

In the present study, the observed interaction between birth satisfaction (BSS-R) and perceived social support (MSPSS) in predicting breastfeeding self-efficacy (BSES-SF) is consistent with Albert Bandura's theoretical framework regarding the sources and mechanisms of self-efficacy (7, 21). According to this theory, beliefs about personal competence emerge through a dynamic interplay between individual experiences and environmental factors, including social support and social persuasion. Direct experiences, such as subjectively perceived birth outcomes, represent a source of self-efficacy; however, their influence is not universal or constant but depends on the social context in which individuals function. The present findings indicate that the strength of the correlation between birth satisfaction and breastfeeding self-efficacy varies across levels of perceived social support.

Furthermore, it is consistent with previous literature that both birth-related experiences and perceived social support have been shown to contribute to breastfeeding self-efficacy. Individual factors—including mode of delivery, early breastfeeding difficulties and subjective perceptions of the birth as traumatic or positive—can shape a mother's initial confidence in her ability to breastfeed (15, 32, 33). At the same time, contextual resources, particularly perceived social support from partners, family and health professionals, have been regularly linked to higher self-efficacy across diverse populations and settings (33–35). Although the specific predictors and theoretical frameworks vary across studies, the present findings align with this broader evidence that both individual and social factors jointly shape maternal breastfeeding confidence. Nonetheless, the conducted moderation analysis revealed that this relationship is non-linear. Our results indicate that the influence of birth satisfaction—a key dimension of the birth experience—on breastfeeding self-efficacy is not uniform but depends on the level of social support: as perceived social support increases, the strength of this association progressively weakens and ultimately reverses at high levels of support. It follows from this finding that when social support is strong, mothers may rely less on their birth satisfaction as a basis for evaluating their breastfeeding competence, potentially drawing on emotional reassurance, practical assistance and external validation instead. Such an interpretation is consistent with Bandura's framework, in which self-efficacy is shaped not only by mastery experiences, but also by social verbal persuasion and an individual's emotional/physiological state; in the breastfeeding context, partner and health-professional attitudes constitute crucial forms of social influence (21, 22, 36). Moreover, empirical data show that perceived social support is a strong independent predictor of BSES-SF and is positively associated with coping/competence, while depressive, anxiety and stress symptoms are inversely related to breastfeeding self-efficacy. This finding suggests that high support may buffer negative effects and provide alternative cues for competence appraisal (11, 36).

The discussed pattern reveals a more nuanced interplay than a simple compensatory model would predict. At low levels of perceived social support, birth satisfaction was not significantly connected with breastfeeding self-efficacy. This null association suggests that, in the absence of external validation, a woman's subjective evaluation of her birth does not automatically translate into higher or lower breastfeeding confidence; the two dimensions appear to operate relatively independently when interpersonal resources are scarce. As perceived social support increased to average and high levels, the relationship became significantly inverse. This counterintuitive result may reflect a psychological shift in the sources from which women derive their breastfeeding confidence. Across diverse cultural settings, higher social support has been consistently linked to higher breastfeeding self-efficacy, with emotional encouragement, practical assistance and verbal persuasion acting as significant reinforcing mechanisms (19, 33, 34, 37). When such robust external support is available, mothers may rely less on their birthing experience as a benchmark for their capabilities; instead, their self-efficacy is likely anchored in the ongoing reassurance provided by their social network. In line with this view, women's beliefs about their maternal capabilities appear to depend more strongly on personal birthing experiences in situations of limited support (38), and less favorable or stressful childbirth experiences may have greater impact on breastfeeding self-efficacy in the absence of adequate social resources. Conversely, at higher levels of perceived social support, breastfeeding self-efficacy appeared less closely linked to birth satisfaction. This pattern may further indicate that well-supported mothers draw on emotional encouragement, practical assistance and external reassurance from their social network when evaluating their breastfeeding confidence, although the cross-sectional design does not allow this interpretation to be tested causally (11, 39, 40). High levels of social support have been consistently associated with increased breastfeeding self-efficacy and better early lactation outcomes, including timely initiation and sustained nursing. Although mothers of pre-term infants were not included in the present research, in other studies, it has been shown that associations between social support and breastfeeding self-efficacy may be particularly pronounced in such vulnerable groups, suggesting that support-related factors should be examined in future trials across different clinical populations (11, 41). Notably, it has not yet been directly tested whether social support moderates the link between birth satisfaction and breastfeeding self-efficacy, although related moderation effects have been observed in general parenting self-efficacy (42). The present study thus provides the first empirical evidence for this specific conditional relationship, suggesting that strengthening social support may be particularly beneficial when birth satisfaction is low, while for well-supported mothers, breastfeeding confidence can develop independently of the childbirth experience.

Although statistically significant, the overall model explained only a modest proportion of variance in breastfeeding self-efficacy (*R*^2^ = 0.029), and the interaction term itself accounted for a small increment (Δ*R*^2^ = 0.0107). This is not unexpected, given the multifactorial nature of breastfeeding self-efficacy, which is shaped by a broad range of biological, psychological, clinical and systemic factors beyond those included in the present model. In comparable studies, social support and related constructs have typically explained greater shares of variance when analyzed alongside more proximal predictors such as depression, sleep quality and early breastfeeding difficulties (33–35, 43). The modest effects observed here may therefore partly reflect the omission of such variables from the analysis. Additionally, the relatively high and homogeneous levels of social support in the present sample likely restricted variance in the moderator, potentially attenuating the interaction effect. Consequently, the identified moderation pattern may be statistically reliable, its individual-level predictive power is limited. The clinical and public health implications should therefore be viewed as contributing to a broader, multi-component support strategy rather than as standalone solutions. The interpretation of these implications should also take the composition of the study sample into account. Due to recruitment being conducted online and the sample including a high proportion of highly educated women with generally favorable socioeconomic conditions, the findings may primarily reflect the experiences of mothers with higher health literacy, better digital access and greater engagement in parenting or breastfeeding-related online communities. Such characteristics may be associated with greater awareness of available support, more active help-seeking and higher baseline breastfeeding confidence. Conversely, mothers with lower educational attainment, limited internet access, fewer material resources or weaker support networks may have been underrepresented. Thus, the observed correlations should not be assumed to represent the full population of Polish mothers, particularly those facing greater social or structural barriers to breastfeeding.

The absence of statistically significant associations for maternal age, parity, partner encouragement, pregnancy complications and mode of birth should be interpreted with caution but this does not necessarily indicate that these factors are unimportant for breastfeeding self-efficacy. Several explanations are possible. First of all, as noted above, the relative homogeneity of the sample in terms of education and socioeconomic status may have reduced variability in some predictors. Secondly, several covariates were measured using broad or dichotomous categories, such as vaginal vs. cesarean birth, presence vs. absence of pregnancy complications, or partner encouragement vs. no encouragement. Such coding may not fully capture clinically relevant differences in severity, timing, quality or subjective meaning of these experiences. Additionally, the influence of these variables may be indirect rather than direct. For example, mode of birth or pregnancy complications may affect breastfeeding self-efficacy through birth satisfaction, early skin-to-skin contact or breastfeeding difficulties, maternal mental health or professional lactation support. Similarly, parity may not adequately reflect previous breastfeeding experience, which was not assessed in detail. Therefore, the lack of significant independent effects in the adjusted model should be understood as specific to the present sample and analytical approach rather than as evidence that these variables have no relevance for breastfeeding confidence. Bearing these limitations in mind, the broader significance of the present findings lies in their consistency with the understanding that breastfeeding success depends on a multi-source support ecosystem.

Such an associative pattern can be considered in the context of a broader understanding of breastfeeding success, which relies on a multi-source support ecosystem. A mother's ability to initiate and sustain breastfeeding is increasingly seen as dependent not just on her individual circumstances, but also on a network of knowledge, skills and systemic support Family and friends form a part of this network. Their emotional and practical support, strengthened through prenatal education and tailored postnatal guidance, has been shown to be crucial for breastfeeding outcomes, especially among vulnerable groups. Other vital pillars of this system include healthcare professionals, community resources and supportive workplace policies (41, 44). Our finding—perceived social support modifies the impact of birth satisfaction—highlights the practical importance of this holistic framework. It suggests that different components of this support network—whether partner education, peer-support programs or professional lactation counseling—may be relevant to breastfeeding confidence and should be further examined as potential sources of efficacy-related support, particularly among mothers reporting less satisfactory birth experiences. From the perspective of public health, these results underscore the significance of strengthening social support systems for postpartum women, particularly those who report negative or challenging birthing experiences reflected in low birth satisfaction. Interventions aimed at enhancing partner involvement, peer support and professional lactation guidance may aid mitigation regarding the potential adverse effects of difficult childbirth on breastfeeding self-efficacy. Moreover, early identification of women with low perceived social support may allow targeted support strategies to be implemented during the perinatal period.

### 4.1 Study limitations and strengths

Several strengths and limitations of this study should be acknowledged. The main strengths include the relatively large sample size, which provided adequate statistical power for moderation analyses, and the integration of perinatal, sociodemographic as well as psychosocial variables within a single analytical framework. The use of standardized and validated instruments (BSES-SF, MSPSS and BSS-R) strengthens the reliability of the measurements. In addition, the inclusion of both primiparous and multiparous women and participants from rural and urban areas enhances the relevance of the findings.

However, several limitations must be considered. First of all, the cross-sectional design precludes causal inference and limits conclusions regarding temporal or directional relationships among birth satisfaction, social support and breastfeeding self-efficacy. Therefore, the observed moderation effect should be interpreted as a conditional statistical association rather than evidence of perceived social support changing, buffering or compensating for the effect of birth satisfaction on breastfeeding self-efficacy.

Secondly, all the variables were assessed via self-report, which introduces the possibility of recall and social desirability biases, particularly for perinatal experiences and breastfeeding-related behaviors. Although restricting eligibility to mothers of infants aged ≤24 months may have reduced long-term recall error, retrospective reporting of childbirth experience and early postpartum practices remains a potential source of measurement inaccuracy.

Thirdly, the participants were recruited online, which may have resulted in selection bias. Open online recruitment tends to favor women with higher digital literacy, greater health awareness and better socioeconomic circumstances. The sample also showed an overrepresentation of women with higher education compared to national statistics for women of reproductive age in Poland (45). This pattern is common in studies using open online recruitment, as such methods tend to attract individuals being more familiar with digital technologies, having higher health awareness and demonstrating more active engagement in parenting communities. Therefore, the results should be interpreted with caution and cannot be directly generalized to the entire population of Polish mothers, particularly to those with lower educational attainment and limited internet access. This selection bias may have influenced the results by restricting variability in key psychosocial and socioeconomic characteristics. In particular, women with higher education and more favorable living conditions may have had greater access to breastfeeding information, professional advice and informal support networks, which could affect both perceived social support and breastfeeding self-efficacy. Consequently, the challenges experienced by mothers with fewer resources, lower health literacy, limited internet access or weaker social support may be underestimated in the current study. The moderation pattern observed in this sample should therefore be interpreted as context-specific and requires confirmation in more socioeconomically diverse and population-based samples.

Moreover, current pregnancy status at the time of data collection was not assessed. Although survey instructions explicitly oriented responses towards the most recent birth and breastfeeding experience, it is possible that some participants became pregnant again. In future studies, current pregnancy should be controlled for to definitively exclude any potential confounding.

Additional limitations include the absence of several potentially relevant factors, such as detailed measures of early breastfeeding difficulties, professional lactation support, maternal mental health beyond perceived social support, as well as infant-related clinical variables (e.g., neonatal complications or Neonatal Intensive Care Unit admissions). These unmeasured factors may have influenced breastfeeding self-efficacy and could partially account for observed associations. Furthermore, the exclusion of pre-term deliveries from the study sample limits generalizability of the findings to mothers of pre-term infants—a group in which both birth satisfaction and breastfeeding self-efficacy may be shaped by additional medical and psychological factors not captured in the present analysis. Similarly, the exclusion of mothers who exclusively formula-fed during the first six months limits the applicability of the findings to women with at least some breastfeeding experience after birth. This group may differ from breastfeeding mothers in terms of medical, psychological, social or structural barriers to breastfeeding, and these determinants would require a separate analytical approach.

Finally, although a significant moderation effect was identified, the proportion of explained variance was modest, suggesting that breastfeeding self-efficacy is shaped by a broader constellation of biological, psychological and contextual influences not fully captured in the current model. Therefore, the findings should be interpreted with caution.

Longitudinal designs should be employed in further research to clarify causal pathways and examine potential mediators, such as early lactation challenges and postpartum mental health. More comprehensive assessment of clinical and environmental factors may further refine the understanding of how birth satisfaction, alongside other dimensions of the childbirth experience, and social support jointly shape breastfeeding self-efficacy.

## 5 Conclusions and implications

In the present study, it is shown that both birth satisfaction and perceived social support are associated with breastfeeding self-efficacy; however, social support significantly moderates this relationship. In the present cross-sectional model, the correlations between birth satisfaction and breastfeeding self-efficacy differed according to the level of perceived social support. At higher levels of support, breastfeeding self-efficacy appeared less closely linked to birth satisfaction, which may reflect the relevance of emotional reassurance and practical assistance. Nonetheless, this interpretation should be tested in longitudinal research. The achieved findings highlight the important role of social support in shaping maternal breastfeeding confidence. Routine assessment of breastfeeding self-efficacy and perceived support in perinatal care may help identify women at risk of low confidence. Interventions involving partners, peer support and early access to lactation counseling may be particularly beneficial for mothers reporting limited support or challenging birth experiences. At the public health level, strengthening family and community support systems alongside promoting supportive maternity care may contribute to improved breastfeeding outcomes.

## Data Availability

The raw data supporting the conclusions of this article will be made available by the authors, without undue reservation.
